# Galvanic Replacement Synthesis of Metal Nanostructures: Bridging the Gap between Chemical and Electrochemical Approaches

**DOI:** 10.1021/acs.accounts.3c00067

**Published:** 2023-03-26

**Authors:** Haoyan Cheng, Chenxiao Wang, Dong Qin, Younan Xia

**Affiliations:** # School of Materials Science and Engineering, Henan University of Science and Technology, Luoyang 471023, P. R. China; † School of Chemistry and Biochemistry, Georgia Institute of Technology, Atlanta, Georgia 30332, United States; ‡ School of Materials Science and Engineering, Georgia Institute of Technology, Atlanta, Georgia 30332, United States; § The Wallace H. Coulter Department of Biomedical Engineering, Georgia Institute of Technology and Emory University, Atlanta, Georgia 30332, United States

## Abstract

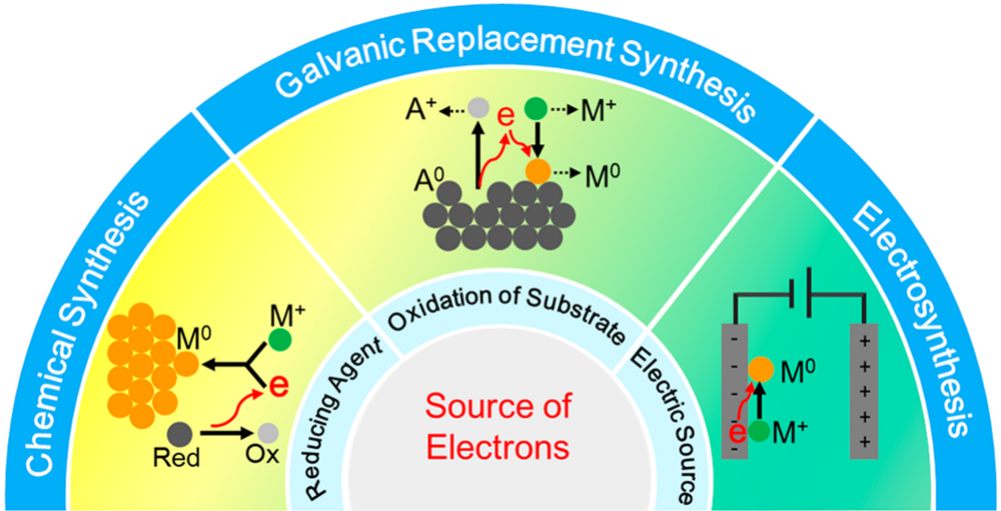

Galvanic replacement synthesis involves oxidation and dissolution of atoms from a substrate while the salt precursor to another material with a higher reduction potential is reduced and deposited on the substrate. The driving force or spontaneity of such a synthesis comes from the difference in reduction potential between the redox pairs involved. Both bulk and micro/nanostructured materials have been explored as substrates for galvanic replacement synthesis. The use of micro/nanostructured materials can significantly increase the surface area, offering immediate advantages over the conventional electrosynthesis. The micro/nanostructured materials can also be intimately mixed with the salt precursor in a solution phase, resembling the setting of a typical chemical synthesis. The reduced material tends to be directly deposited on the surface of the substrate, just like the situation in an electrosynthesis. Different from an electrosynthesis where the two electrodes are spatially separated by an electrolyte solution, the cathodes and anodes are situated on the same surface, albeit at different sites, even for a micro/nanostructured substrate. Since the oxidation and dissolution reactions occur at sites different from those for reduction and deposition reactions, one can control the growth pattern of the newly deposited atoms on the same surface of a substrate to access nanostructured materials with diverse and controllable compositions, shapes, and morphologies in a single step. Galvanic replacement synthesis has been successfully applied to different types of substrates, including those made of crystalline and amorphous materials, as well as metallic and nonmetallic materials. Depending on the substrate involved, the deposited material can take different nucleation and growth patterns, resulting in diverse but well-controlled nanomaterials sought for a variety of studies and applications.

In this Account, we recapitulate our efforts over the past two decades in fabricating metal nanostructures for a broad range of applications by leveraging the unique capability of galvanic replacement synthesis. We begin with a brief introduction to the fundamentals of galvanic replacement between metal nanocrystals and salt precursors, followed by a discussion of the roles played by surface capping agents in achieving site-selected carving and deposition for the fabrication of various bimetallic nanostructures. Two examples based on the Ag–Au and Pd–Pt systems are selected to illustrate the concept and mechanism. We then highlight our recent work on the galvanic replacement synthesis involving nonmetallic substrates, with a focus on the protocol, mechanistic understanding, and experimental control for the fabrication of Au- and Pt-based nanostructures with tunable morphologies. Finally, we showcase the unique properties and applications of nanostructured materials derived from galvanic replacement reactions for biomedicine and catalysis. We also offer some perspectives on the challenges and opportunities in this emerging field of research.

## Key References

- ChengH.; WangC.; LyuZ.; ZhuZ.; XiaY.Controlling the Nucleation and Growth of Au on *a*-Se Nanospheres to Enhance Their Cellular Uptake and Cytotoxicity. J. Am. Chem. Soc.2023, 145, 1216–12263662198810.1021/jacs.2c11053.^1^*This work demonstrated the synthesis of Au nanoparticles from the surface of amorphous Se nanospheres through a galvanic replacement reaction, where the nucleation and growth pattern of Au could be manipulated by controlling the reaction kinetics*.
- ChengH.; CaoZ.; ChenZ.; ZhaoM.; XieM.; LyuZ.; ZhuZ.; ChiM.; XiaY.Catalytic System Based on Sub-2 nm Pt Particles and Its Extraordinary Activity and Durability for Oxygen Reduction. Nano Lett.2019, 19, 4997–50023130508610.1021/acs.nanolett.9b01221.^2^*This work demonstrated the in situ synthesis of sub-2 nm Pt particles on a commercial carbon support by leveraging the galvanic replacement reaction between a Pt^II^ precursor and a thin film of amorphous Se preformed on the carbon support*.
- AhnJ.; WangD.; DingY.; ZhangJ.; QinD.Site-Selective Carving and Co-Deposition: Transformation of Ag Nanocubes into Concave Nanocrystals Encased by Au-Ag Alloy Frames. ACS Nano2018, 12, 298–3072925766410.1021/acsnano.7b06353.^3^*This work demonstrated the use of Cl^–^ ions as a capping agent for the Ag{100} facets to initiate galvanic replacement exclusively from the side faces of Ag nanocubes, leading to the formation of Au–Ag alloy nanoframes*.
- ZhangH.; JinM.; WangJ.; LiW.; CamargoP. H. C.; KimM. J.; YangD.; XieZ.; XiaY.Synthesis of Pd-Pt Bimetallic Nanocrystals with a Concave Structure through a Bromide-Induced Galvanic Replacement Reaction. J. Am. Chem. Soc.2011, 133, 6078–60892143859610.1021/ja201156s.^4^*This work demonstrated the use of Br^–^ ions as a capping agent selective toward the Pd{100} facets to initiate galvanic replacement exclusively from the side faces of Pd nanocubes for the generation Pd–Pt bimetallic nanostructures with a concave surface*.

## 1 Introduction

Metal nanostructures have attracted steadily growing interest owing to their fascinating properties for applications in catalysis, plasmonics, sensing, and biomedicine. The last two decades has witnessed the development of methods for synthesizing metal nanostructures with controlled compositions, sizes, shapes, morphologies, and internal structures. Typically, the synthesis involves the reduction of a salt precursor to metal atoms upon receiving electrons, followed by a series of nucleation and growth events to produce metal nanostructures. In the setting of a wet chemical synthesis, the electrons are supplied by a reducing agent (i.e., an electron donor) homogeneously mixed with the precursor throughout the reaction medium. The precursor can be reduced to metal atoms in the solution phase, followed by their deposition onto the surface of a growing particle.^5^ Alternatively, the precursor can adsorb onto the surface of a growing particle, followed by reduction and incorporation into the nanostructure through an autocatalytic process. With the assistance of a colloidal stabilizer and capping agent, the resultant nanostructures can stay in a stable suspension while evolving into different shapes or morphologies along with size enlargement. As constrained by the balance of electrons, a stoichiometric amount of the reducing agent must be added, in one shot or dropwise, to reduce all the precursor in the reaction solution. For dropwise titration, the frequency is defined by the duration of time between two adjacent drops, and it is controlled by the injection rate. For an electrosynthesis, in contrast, the electrons are supplied by an external electrical current to the cathode where the precursor is reduced. In this case, redox transformations are achieved via the coupling of cathodic reduction and anodic oxidation, with the number of electrons involved being controlled by the current density and duration of time.^6^ Both direct current and pulse power supplies can be used in electrosynthesis. In the latter case, the “pulsed” supply of electrons is controlled by the electric current, which can change periodically with a widely adjustable frequency. Any delay time can be inserted between two successive current pulses, allowing for controllable deposition parameters, such as frequency and deposition rate at the deposition interface. The metal cations are reduced to atoms on the surface of the cathode for the deposition of a polycrystalline film through heterogeneous nucleation.^7^ Under certain conditions, however, the resultant atoms can also diffuse into the solution phase to generate metal nanostructures with tunable sizes and morphologies with the assistance of a colloidal stabilizer.^8^ For example, Wang et al. synthesized Au nanorods using an electrochemical method, where Au and Pt plates served as the anode and cathode, respectively.^8^ During the synthesis, the Au plate at the anode was electrically oxidized to Au^III^ and released into the electrolyte solution, and then reduced back to Au atoms. As such, there was no need to directly add Au^III^ precursor into the electrolyte.

Situated between chemical synthesis and electrosynthesis, galvanic replacement offers an alternative approach to the preparation of metal nanostructures. It relies on the spontaneous redox reaction between the atoms of a substrate and the salt precursor to a less reactive metal.^4^ If the substrate is supplied in the form of micro/nanostructured materials, the salt precursor can be intimately mixed with the substrate in a solution phase and reduced to atoms on the surface of the substrate by the electrons originating from the substrate, in a fashion similar to a chemical synthesis. The resultant atoms tend to grow into nanostructures right on the surface of the substrate because of a lower energy barrier to heterogeneous nucleation than homogeneous nucleation. The galvanic replacement reaction can also be split into two half reactions: (i) oxidation and dissolution of the substrate at the anode and (ii) reduction of the salt precursor and deposition of the resultant metal atoms at the cathode. This feature is similar to what is involved in an electrosynthesis. In fact, the substrate involved in a galvanic replacement synthesis can be considered as an ensemble of multiple cathodes and anodes. The spontaneity of a galvanic replacement reaction is determined by the difference in potential between the two half reactions.^9^ Both galvanic replacement and chemical reduction can be set to occur simultaneously if an additional reducing agent is added into the reaction solution, further affecting the composition and morphology of the product.^3,10^ It is necessary to add a colloidal stabilizer if one wants the final product to stay as a colloidal suspension.

Table 1 shows a comparison of the major characteristics of these three synthetic methods, demonstrating that galvanic replacement is in a unique position to bridge the gap between chemical and electrochemical approaches. As a major advantage over electrosynthesis, the substrates involved in a galvanic replacement can take the form of nanocrystals to significantly increase the surface area. In fact, galvanic replacement synthesis can be considered as a special case of electrosynthesis, where there is no physical separation between the cathode and anode. However, since the oxidation and reduction reactions still occur at different sites on the same surface of a nanocrystal, it is feasible to tailor the final morphology of the product by controlling the site of deposition while mitigating the issue of reagent trafficking.^3,4^

**Table 1:** Comparison of the Major Features of Chemical Synthesis, Galvanic Replacement Synthesis, and Electrosynthesis of Metal Nanostructures in Solution Phase

	chemical synthesis	galvanic replacement synthesis	electrosynthesis
source of electrons	reducing agent	substrate	external electrical current
supply of electrons	continuous (one-shot) or pulsed (dropwise)	continuous (one-shot) or pulsed (dropwise)	continuous or pulsed
number of electrons	determined by the amount of reductant	self-limited by the number of atoms in the substrate	determined by the current density and duration of time
reduction pathway	solution and/or surface reduction	surface reduction	surface reduction
nucleation behavior	homogeneous and/or heterogeneous	heterogeneous	homogeneous or heterogeneous
control of reduction rate constant	difference in reduction potential between the precursor and reducing agent	difference in reduction potential between the redox pairs	externally applied potential
mixing between the precursor and electron donor	intimately mixed in a solution phase	can be intimately mixed in a solution phase	in physical contact through an interface
reducing agent	needed	not needed	not needed
precursor	needed	needed	not necessary
colloidal stabilizer	needed	not necessary	not necessary
capping agent	needed	not necessary	not necessary
temperature	over a broad range	over a broad range	room temperature
scale-up production	continuous flow	continuous flow	still challenging

## 2 Galvanic Replacement Synthesis

**Figure 1:**
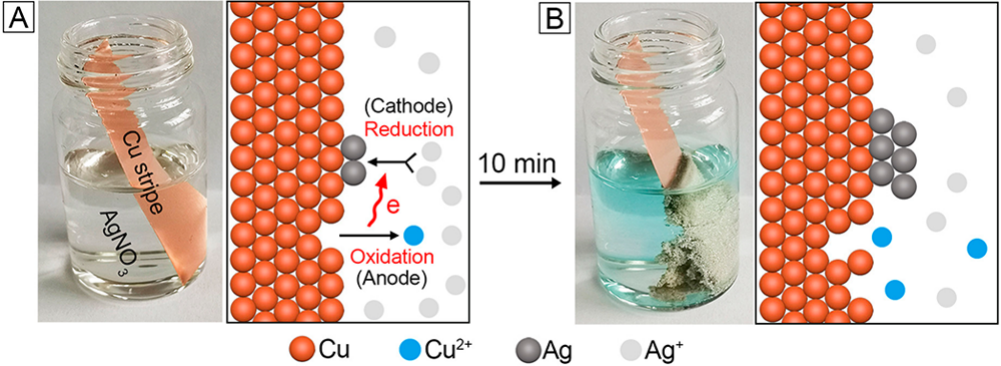
Photographs of a glass vial containing AgNO_3_ solution immediately (A) after the insertion of a Cu strip and (B) after the reaction had proceeded for 10 min. The atomic models illustrate the redox reactions involved in the galvanic replacement between the surface of the Cu strip and the Ag^+^ ions.

Figure 1 shows a typical example of galvanic replacement synthesis, which is conducted by simply inserting a Cu strip into an aqueous AgNO_3_ solution. In this synthesis, the Cu atoms are oxidized to Cu^2+^ and dissolved into the solution. The resultant electrons are captured by the Ag^+^ cations to generate Ag atoms through a reduction process. The newly formed Ag atoms are directly deposited on the surface of the Cu strip, loosely assembled into a porous film on the surface of the Cu strip. The reactions involved in this synthesis can be summarized as the following:^9^Anode half reaction:
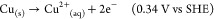
1Cathode half reaction:
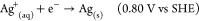
2Combined reaction:
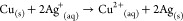
3

In principle, galvanic replacement synthesis should work equally well for substrates with different dimensions, including bulk samples (Figure 1) and those on the micro/nanoscale. The only requirement is that the difference in reduction potential between the substrate and salt precursor must be negative. In practice, the actual reduction potential is dependent on many experimental parameters, including temperature as well as coordination ligands and concentrations of relevant ions. We have shown that I^–^ ions could be used to lower the reduction potential of Pd^2+^/Pd pair (Pd^2+^/Pd, 0.95 V vs SHE; PdI_4_^2–^/Pd, 0.18 V vs SHE), reversing the direction of galvanic replacement between Pd nanocubes and Ru^III^ ions in an aqueous solution containing NaI.^11^ In addition to coordination ligands, the precursor concentration also has a direct impact on the reduction potential, as reflected in the Nernst equation. We have demonstrated that the galvanic replacement reaction between Rh^III^ precursor and Ag nanocubes could only be triggered to form Rh islands when the precursor concentration reached a certain level.^12^Table 2 summarizes the pairs of metals that have been reported for galvanic replacement synthesis of metal nanostructures.

**Table 2:** Pairs of Metals That Have Been Reported for the Synthesis of Nanostructures through Galvanic Replacement

substrate	deposited metal
Ag	Rh,^12^ Pd,^13^ Ir,^14^ Pt,^13^ Au^15^
Pd	Au,^16^ Ir,^17^ Pt,^4^ Rh,^18^ Ru^11^
Cu	Ru,^19^ Rh,^19^ Os,^19^ Ir,^19^ Ag,^20^ Pt,^21^ Pd,^21^ Au^21^
Co	Ru,^22^ Rh,^23^ Ag,^24^ Pd,^25^ Ir,^26^ Pt^27^

As a unique feature of galvanic replacement synthesis, the overall shape or morphology of the product tends to replicate that of the nanocrystal substrate while it can contain a hollow interior originally occupied by the substrate and pores corresponding to the sites for oxidation reaction (i.e., anodes). The substrate can be either metallic (e.g., Ag,^13^ Pd,^4^ or Cu^19^) or nonmetallic (e.g., Se,^10^ Te,^28^ Si,^29^ or Mn_3_O_4_^30^). Previous review articles on this synthetic method have focused on the morphological evolution of metallic substrates and the hollow or frame-like products derived from them.^31−33^ None of them covers nonmetallic substrates. This Account highlights our recent efforts in extending galvanic replacement synthesis from metallic to nonmetallic substrates for the fabrication of metal nanostructures with controlled compositions, shapes, and morphologies. We start with metallic substrates that can be supplied in the form of nanocrystals with controlled shapes, with a focus on how capping agents can be selected to control the initial sites for galvanic replacement. We then illustrate the differences in both nucleation and growth behaviors when switching to nonmetallic substrates. Finally, we highlight unique properties and applications enabled by the metal nanostructures synthesized through galvanic replacement in the context of biomedicine and electrocatalysis.

## 3 Metallic Substrates

**Figure 2:**
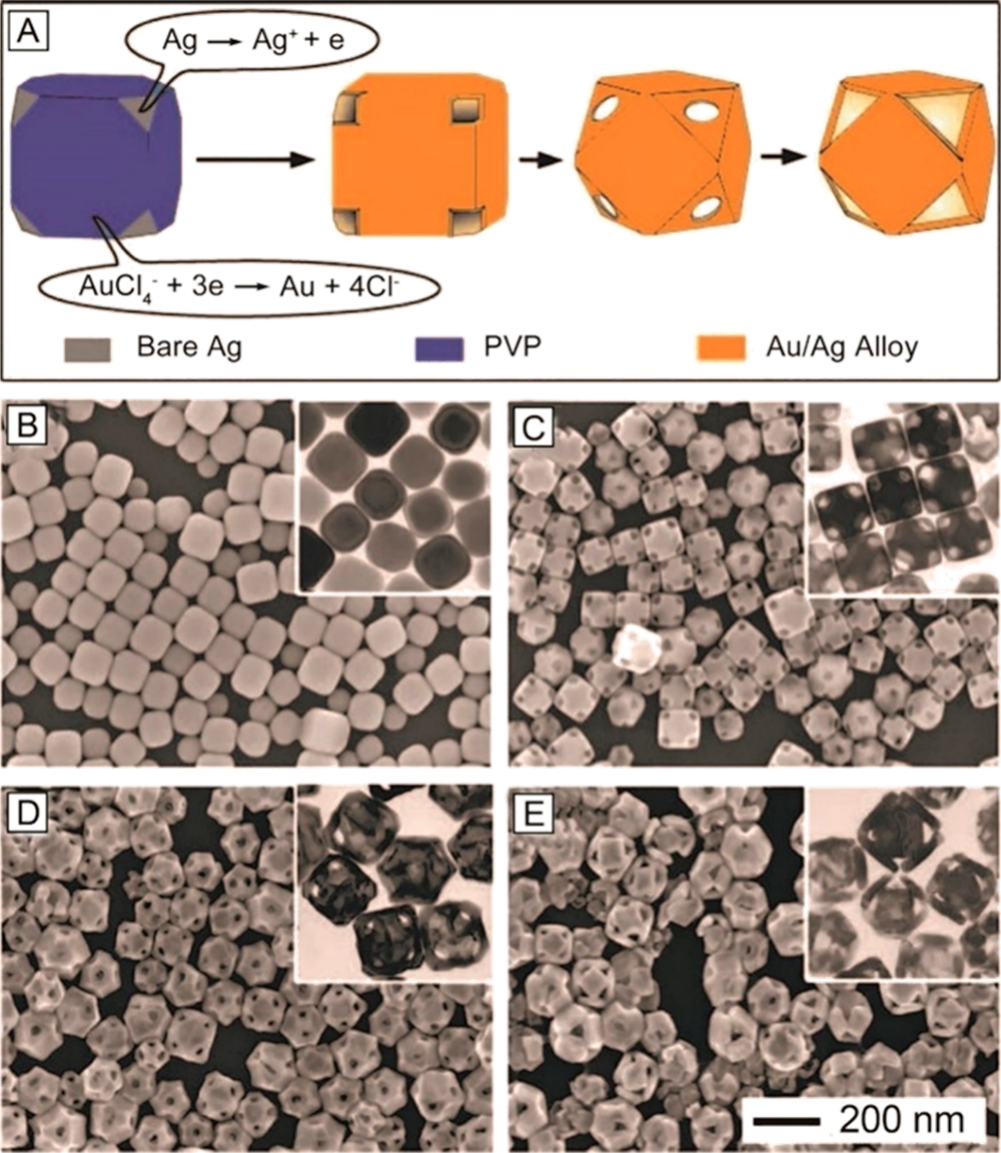
(A) Schematic illustration of the morphological evolution of truncated Ag nanocubes at different stages of a galvanic replacement reaction with HAuCl_4_. (B–E) SEM and TEM images of the nanocubes with truncated corners after reaction with (B) 0, (C) 0.6, (D) 1.6, and (E) 3.0 mL of 0.1 mM HAuCl_4_, respectively. Reproduced with permission from ref (37). Copyright 2006 American Chemical Society.

Our extensive research has demonstrated that hollow nanostructures comprised of various noble metals such as Au, Pd, and Pt could be obtained through galvanic replacement between Ag nanocrystals and a salt precursor to the desired metal.^13,15^ For example, Ag nanocrystals with various shapes could react with HAuCl_4_ to generate Au-based nanocages, nanoframes, and nanorattles in a single step.^34−36^ In the case of Ag nanocubes with sharp corners, the galvanic reaction tends to be randomly initiated from the side faces for the generation of Au-based nanocages with porous walls.^37^ The initial site for galvanic replacement and thus dissolution of substrate can also be controlled by introducing a facet-specific capping agent. For example, when Ag nanocubes with truncated corners were used, the oxidization and dissolution of Ag would be initiated from the eight corners enclosed by {111} facets if the {100} side faces were covered by poly(vinyl pyrrolidone) (PVP).^37^ The initial oxidization sites served as anodes for the constant dissolution of Ag atoms and thus release of electrons. The excellent electric conductivity of Ag allowed the electrons to quickly transport to the {100} facets, which acted as cathodes for the formation of Au atoms. The resultant Au atoms were deposited evenly on each facet because of the same crystal structure and almost identical lattice constant between Ag and Au. With the progression of galvanic replacement, Au–Ag alloy nanocages with a cubic shape and pores at all the corners were formed (Figure 2A). The SEM images in Figure 2B–E show the morphological evolution of truncated Ag nanocubes after reaction with different volumes of HAuCl_4_. The size of the pores was gradually enlarged until the area of the {111} facets could not be increased any further.

**Figure 3:**
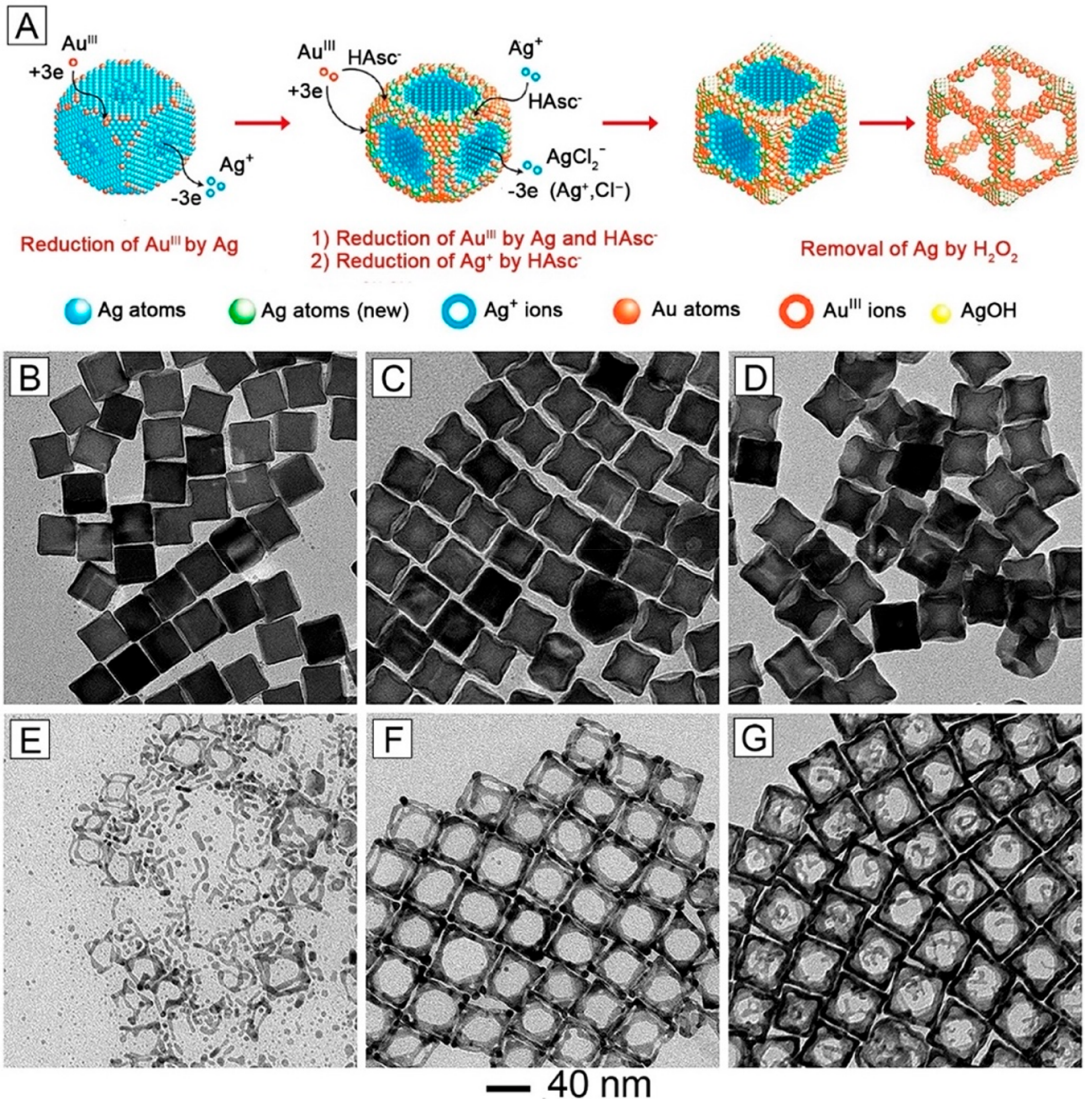
(A) Schematic showing the morphological changes of Ag nanocubes at different stages of galvanic replacement with HAuCl_4_ in the presence of H_2_Asc and CTAC at an initial pH of 11.6. (B–D) TEM images of Ag@Au–Ag concave nanocubes formed after 0.2, 0.8, and 1.6 mL of 0.1 mM aqueous HAuCl_4_ had been added, respectively. (E–G) TEM images of the resultant structures after etching away the residual Ag in the core with aqueous H_2_O_2_. Reproduced with permission from ref (3). Copyright 2018 American Chemical Society.

In another demonstration involving Ag nanocubes, we could initiate the dissolution of Ag atoms from the side faces instead of corners with the assistance of Cl^–^ ions, leading to the formation of Au–Ag alloy nanoframes with pores located on the side faces (Figure 3A).^3^ Specifically, the nanostructures were synthesized by titrating HAuCl_4_ solution into an aqueous mixture containing Ag nanocubes, ascorbic acid (H_2_Asc), NaOH, and cetyltrimethylammonium chloride (CTAC) at an initial pH of 11.6. In the presence of sufficient Cl^–^ and OH^–^, the added HAuCl_4_ was converted to AuCl_4_^–^ without further transformation into AuCl(OH)_3_^–^ and Au(OH)_4_^–^, making it easier to initiate the galvanic replacement reaction with Ag. Like PVP, Cl^–^ ions also selectively bind to Ag{100} facets. Different from PVP, Cl^–^ ions can initiate oxidative etching, resulting in preferential dissolution of Ag from the side faces of a nanocube. Upon the introduction of aqueous HAuCl_4_, the {100} facets acted as anodes for the initiation of galvanic replacement reaction. Meanwhile, due to the presence of H_2_Asc, the AgCl_2_^–^ arising from the oxidation process was reduced together with AuCl_4_^–^ for co-deposition onto the edges and corners. With the progression of galvanic replacement, Ag atoms would be gradually carved away from the side faces, eventually resulting in Ag@Au–Ag nanocubes with pits at side faces (Figure 3B–D). Upon etching of residual Ag from the core with aqueous H_2_O_2_, the concave nanocubes were transformed into Au–Ag alloy nanoframes (Figure 3E–G).

**Figure 4:**
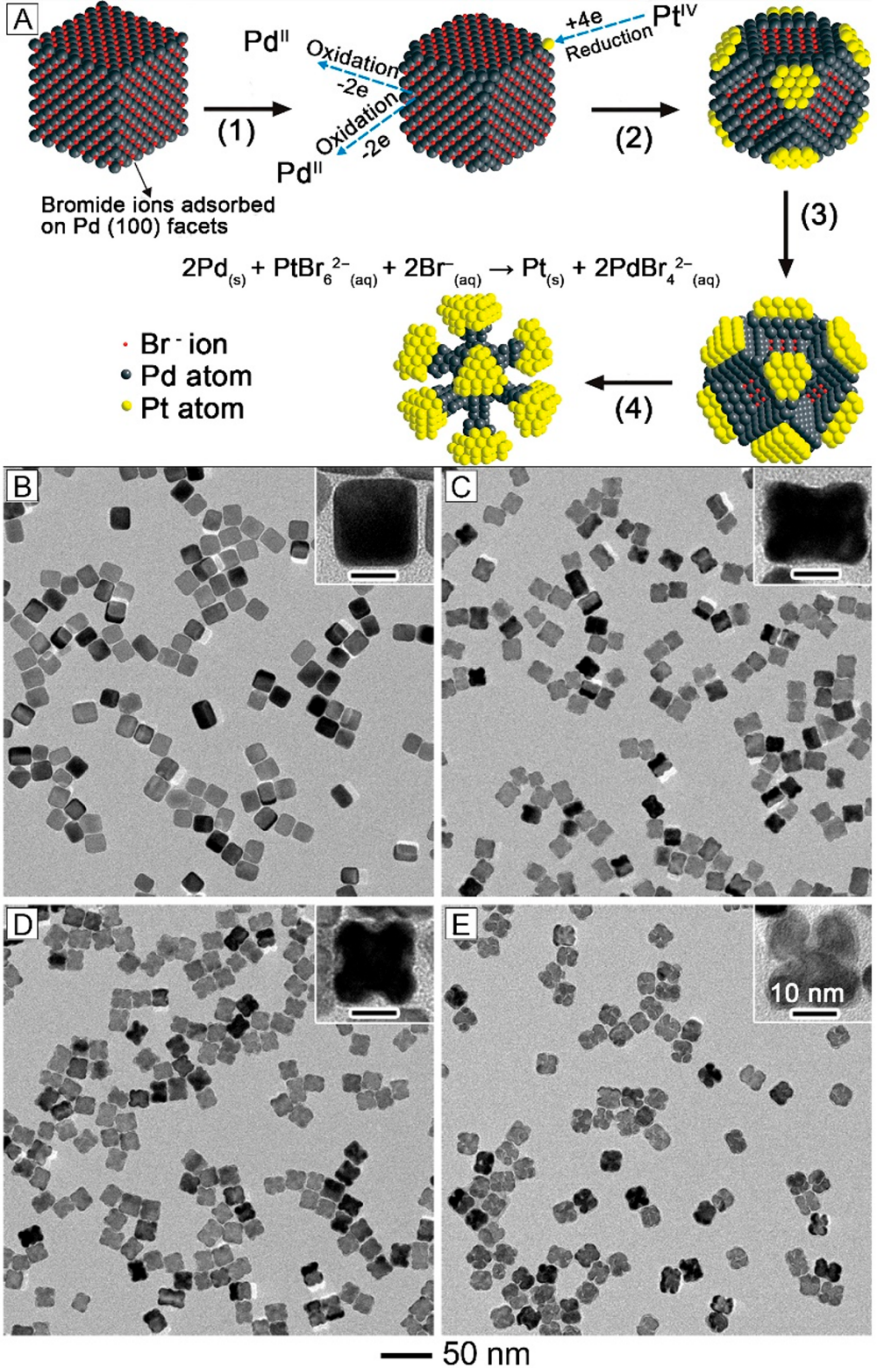
(A) Schematic illustrating the morphological changes of a Pd nanocube at different stages of a galvanic replacement reaction. (B–E) TEM images of Pd–Pt nanostructures prepared via Br^–^-induced reaction between Pd nanocubes and Pt^IV^ at different time points: (B) 0.5, (C) 4, (D) 9, and (E) 20 h. The insets show TEM images of individual nanocrystals at a higher magnification. Reproduced with permission from ref (4). Copyright 2011 American Chemical Society.

In a different system, we demonstrated the synthesis of Pd–Pt nanocrystals with concave side faces via Br^–^-induced galvanic replacement between Pd nanocubes and PtCl_6_^2–^ ions (Figure 4A).^4^ The Br^–^ ions led to the selective initiation of galvanic replacement from the {100} facets of a Pd nanocube due to their oxidative etching capability. When H_2_PtCl_6_ was added into an aqueous mixture containing Pd nanocubes, PVP, and KBr, the PtCl_6_^2–^ complex quickly evolved into PtBr_6_^2–^ through ligand exchange. The Pd{100} facets acted as anodes and were preferentially oxidized while the PtBr_6_^2–^ ions were reduced to Pt atoms and deposited onto the corners (cathodes) of the nanocube. As the reaction proceeded, the {100} facets were gradually oxidized and dissolved together with the deposition of Pt atoms at the corners, resulting in the formation of Pd–Pt concave nanocubes. The products obtained at different stages of a synthesis revealed a transition in morphology from nanocubes to concave nanocubes, and then to octopods with gradually enlarged bumps at the tips after complete removal of the Pd{100} facets (Figure 4B–E).

## 4 Nonmetallic Substrates

**Figure 5:**
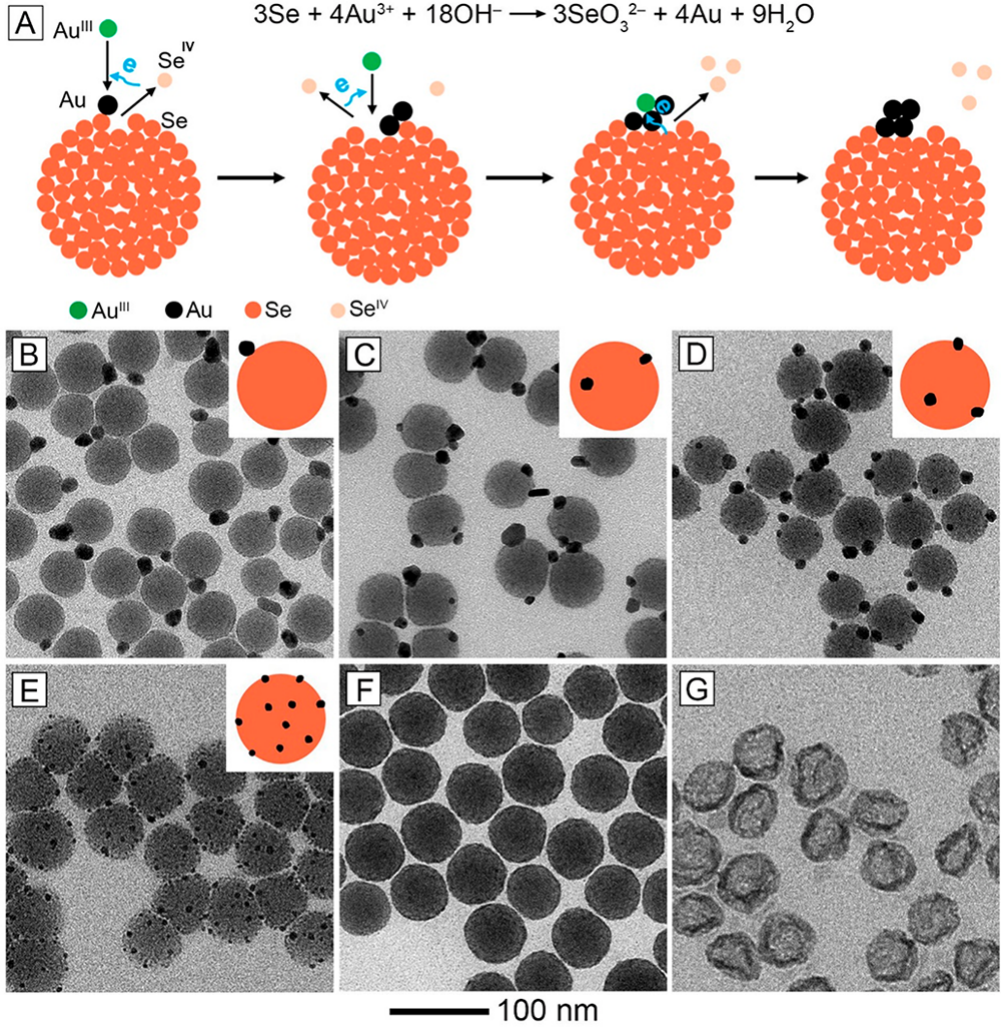
(A) Galvanic replacement between *a*-Se nanosphere and Au^III^ precursor. (B–E) TEM images of Se–Au hybrid nanoparticles obtained at different initial pH values of (B) 8.2, (C) 9.0, (D) 10.0, and (E) 11.0 at room temperature. (F) TEM image of the Se@Au core–shell nanoparticles obtained at 60 °C. (G) TEM image of Au hollow nanoparticles. Insets: the corresponding two-dimensional models. Reproduced with permission from ref (1). Copyright 2023 American Chemical Society.

In addition to those based on metals, we have also explored nonmetallic substrates for galvanic replacement synthesis. In one example, we demonstrated the use of amorphous Se (*a*-Se) nanospheres as a substrate for the facile synthesis of Se–Au hybrid nanoparticles with a variety of morphologies ranging from Janus to core–shell structures.^1^ The key is to control the number of heterogeneous nucleation sites on the surface of an *a*-Se nanosphere by adjusting the kinetics associated with the galvanic replacement reaction. When aqueous HAuCl_4_ is added dropwise into an aqueous suspension of *a*-Se nanospheres and CTAB, Se atoms will be oxidized to Se^IV^ and the released electrons will be captured by Au^III^ to generate Au atoms via a reduction reaction (Figure 5A). Owing to the drastic difference between the substrate and the metal being deposited, the resultant Au atoms favor island growth characterized by nucleation and growth at the original site of formation. This growth pattern is completely different from the case involving a crystalline, metallic substrate such as Ag nanocubes. Because Au and Ag share the same crystal structure with similar lattice constants, the Au atoms can readily diffuse across the surface of the Ag substrate for the creation of a Au-based shell around the substrate. In the case of *a*-Se, once Au nuclei have been formed on the surface of the substrate at the beginning of a synthesis, the Au^III^ ions tend to be reduced to Au atoms on the newly formed Au surface due to the absence of lattice mismatch. As a result, the number of Au nanoparticles formed on each *a*-Se nanosphere will be determined by the number of the initial nucleation sites. In return, the number of initial Au nuclei per *a*-Se nanosphere can be controlled by adjusting the pH of the reaction solution and thereby the reduction rate of the Au^III^ precursor. Specifically, Se–Au hybrid nanostructures with roughly 1, 2, 3, and 10 Au nanoparticles per *a*-Se nanosphere (Figure 5B–E) were obtained when the initial pH was set to 8.2, 9.0, 10.0 and 11.0, respectively. When the reaction temperature was raised from 25 to 60 °C, the reduction of Au^III^ would be significantly accelerated to allow the formation of plentiful Au nuclei on the surface of each *a*-Se nanosphere, yielding a core–shell structure (Figure 5F). During TEM imaging, the Se in the core quickly evaporated upon e-beam irradiation, resulting in the formation of Au hollow nanoparticles (Figure 5G).

**Figure 6:**
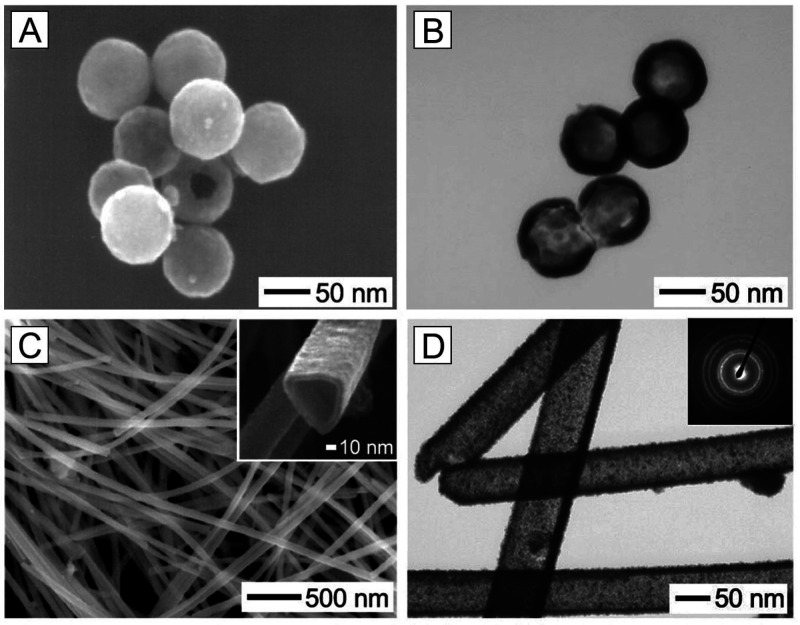
SEM and TEM images of (A, B) Pt hollow nanospheres and (C, D) Pt nanotubes after dissolving the Se in the core with pure hydrazine. Reproduced with permission from ref (10). Copyright 2003 American Chemical Society.

A similar approach was also applied to the synthesis of nanostructures made of other noble metals. For example, using *a*-Se nanospheres and trigonal selenium (*t*-Se) nanowires as the substrates, we have fabricated hollow nanospheres and nanotubes, respectively, comprised of Pt (Figure 6).^10^ Different from the previous example, ethanol instead of water was used as the solvent and an additional reducing agent. In this case, Pt^II^ precursor was reduced *in situ* on the surface of *a*-Se nanospheres or *t*-Se nanowires, followed by their nucleation and growth into a polycrystalline shell. The galvanic replacement between Se substrates and Pt^II^ would continue until the surface of the substrate was completely blocked by Pt. Subsequently, the reduction was dominated by the ethanol, with the thickness of the Pt wall being controlled by the amount of the Pt^II^ precursor. At the end, the remaining Se could be removed through dissolution with pure hydrazine for the generation of Pt hollow nanostructures.

**Figure 7:**
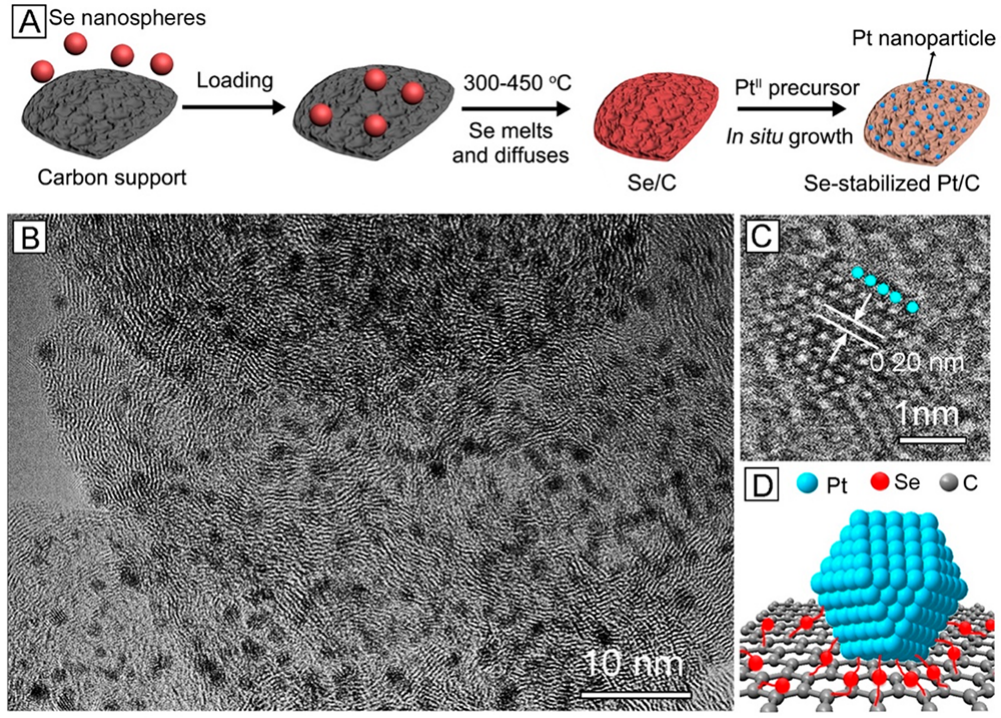
(A) Schematic showing the preparation of a Se-stabilized Pt/C catalyst. (B) TEM and (C) high-resolution TEM image of the Se-stabilized Pt/C catalyst. (D) Schematic showing how to anchor a Pt nanoparticle to the carbon surface through the Pt–Se–C linkage. Reproduced with permission from ref (2). Copyright 2019 American Chemical Society.

In a fashion similar to the conventional electrosynthesis, we were able to grow sub-2 nm Pt particles *in situ* on a commercial carbon support by leveraging the galvanic replacement reaction between a Pt^II^ precursor and a thin film of *a*-Se preformed on a carbon support without using any stabilizer (Figure 7A).^2^ Specifically, *a*-Se nanospheres were deposited on a carbon support, followed by thermal annealing under Ar protection to generate a thin coating on the carbon surface. Upon contacting Pt^II^ precursor, the Se film could serve as reducing agent for the generation of 1.6 nm Pt particles on the carbon support with good dispersion (Figure 7B,C). The average size of the Pt nanoparticles and the mass loading of Pt on the carbon support could both be tuned by varying the temperature used to anneal the *a*-Se nanospheres and thus the thickness of the Se coating. Significantly, we further demonstrated that the residual Se atoms on the surface could serve as a chemical linker to anchor the Pt nanoparticles to the carbon support (Figure 7D), greatly enhancing the stability of the electrocatalyst.

Galvanic replacement synthesis involving nonmetallic substrates has also been reported for other materials, including Si (for Cu, Ag, Au, Pt, Pd, and Rh)^29^ and Te (for Ir,^28^ Rh,^38^ and Ru^39^), for the fabrication of various metal nanostructures. Most of the products were characterized by a rough surface because of the structural mismatch between the nonmetallic substrates and crystalline metals, similar to what was observed for the Se and Pt pair. The morphology of the resultant structures is independent of the capping agent, but it can be tailored by controlling the shape of the substrates and compositions of the reaction solution, as well as the reaction time and temperature. In general, the role of stabilizing agents is to improve the stability of the colloidal suspension.

## 5 Applications

The metal nanostructures prepared using galvanic replacement synthesis have been explored for a variety of applications. Here we focus on their use in controlled release, enhanced cellular uptake, and electrocatalysis.

### 5.1 Au-Based Nanocages for Controlled Release

**Figure 8:**
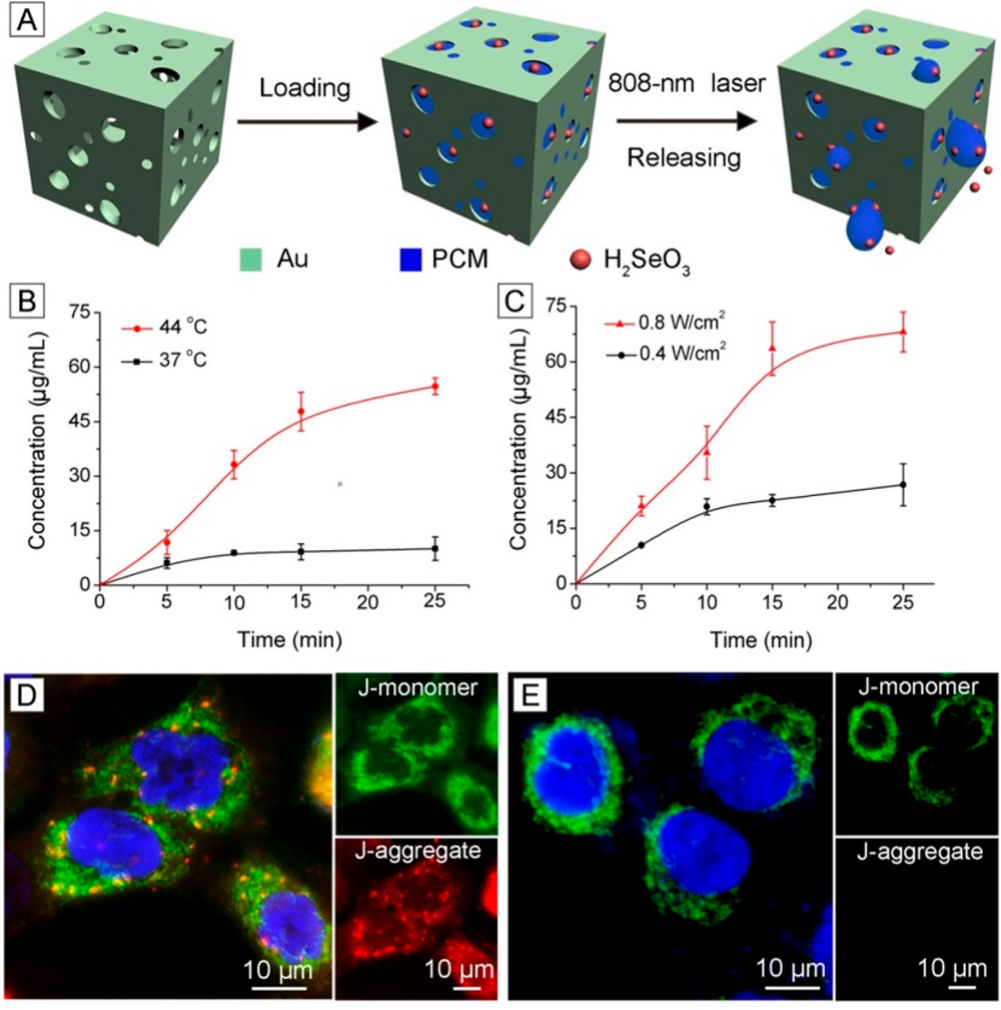
(A) Schematic showing the loading of H_2_SeO_3_ into Au nanocage with a PCM and then on-demand release upon irradiation with an NIR laser. (B and C) Release profiles of the H_2_SeO_3_ (B) at different temperatures and (C) upon irradiation with an 808 nm laser for different periods of time. (D and E) Fluorescence micrographs of the JC-1-stained A549 cells cultured with the H_2_SeO_3_-loaded nanocages (D) before and (E) after irradiation by the laser. Reproduced with permission from ref (42). Copyright 2018 Elsevier.

Galvanic replacement offers a versatile route to Au-based nanostructures with tunable localized surface plasmon resonance (LSPR) properties.^40,41^ When their LSPR peak was tuned into the near-infrared (NIR) region, the nanocages could be used to effectively convert NIR light to heat while allowing deep penetration into soft tissues. The hollow interior and strong photothermal effect of Au-based nanocages make them fascinating carriers for controlled release and drug delivery. Encapsulation of therapeutics in their hollow interiors also enables combination therapy involving both photothermal eradication and chemotherapy. We have demonstrated this concept by encapsulating an anticancer drug (e.g., H_2_SeO_3_, DOX, AIPH, or Ca^2+^) into the Au-based nanocages.^42−45^Figure 8A shows the schematic of a typical example involving the use of H_2_SeO_3_, which was mixed with phase-change material (PCM) with a melting point at 43 °C and then loaded into the cavities of nanocages.^42^ The nanocages could serve as a carrier during cell endocytosis and then as a photothermal transducer to melt the PCM upon irradiation with a NIR laser, triggering the on-demand release of H_2_SeO_3_. The release profile of H_2_SeO_3_ from the nanocages was controlled by the temperature or the power density of the NIR laser (Figure 8B,C). Significantly, the photothermal and chemo-therapies could work synergistically, leading to enhanced eradication of cancer cells. A mechanistic study suggested that the impaired mitochondrial function arising from the ROS generated through combination treatment was responsible for the cell death (Figure 8D).

### 5.2 Se–Au Hybrid Nanoparticles for Enhanced Cellular Uptake

**Figure 9:**
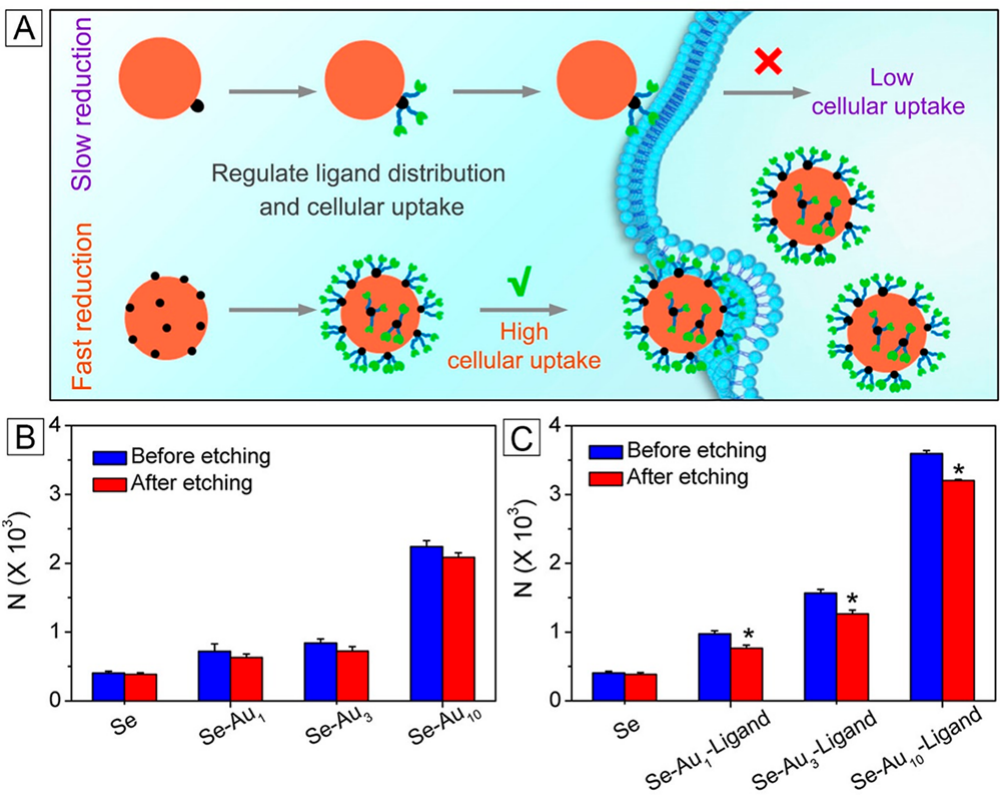
(A) Schematic showing the cellular uptake of Se–Au nanoparticles mediated and controlled by the spatial distribution of targeting ligand. (B and C) Number of Se–Au hybrid nanoparticles internalized per HeLa cell in the (B) absence and (C) presence of targeting ligand. In panels B and C, the nanoparticles remaining on the cell surface could be selectively removed by etching with I_2_/KI solution. Reproduced with permission from ref (1). Copyright 2023 American Chemical Society.

Cellular uptake of nanoparticles is a process pivotal to the development of nanomedicines and related applications. It is mediated and controlled by the interactions between the receptors on the cell membrane and the ligands on the surface of a nanoparticle. The enriched diversity in terms of surface configuration for the Se–Au hybrid nanoparticles shown in Figure 5 offers an opportunity to optimize the spatial distribution of ligands for enhanced cellular uptake and cytotoxicity.^1^ Specifically, the Au patches on the surface of *a*-Se nanoparticles can serve as a platform for the facile and reliable attachment of a targeting ligand such as folic acid-terminated thiol poly(ethylene glycol). Figure 9A shows a schematic illustration of the mechanistic details involved in the cellular uptake of Se–Au nanoparticles as mediated by the ligand distribution. The Se–Au hybrid nanoparticles with more Au nanoparticles on the surface gave a much higher efficiency of endocytosis, which could be further increased by conjugating a targeting ligand to the Au patches (Figure 9A,B). This trend indicates that a larger number of Au nanoparticles on the surface of each *a*-Se nanosphere, and thereby an increased density of targeting ligands, could significantly promote a stronger ligand–receptor interaction for the enhancement of cellular uptake. The different efficiencies in cellular uptake might result from their differences in terms of receptor availability, ligand–receptor interaction, and the equilibrium condition between the ligand distribution and cell perception. The internalized nanoparticles led to dysfunction of mitochondria and eventually cell death.

### 5.3 Pt-Based Catalysts toward Oxygen Reduction

There is an urgent demand to develop Pt-based catalysts with augmented activity and durability toward oxygen reduction, a reaction key to the operation of proton–exchange membrane fuel cells. To this end, galvanic replacement offers an attractive route to the production of Pt-based catalysts with desired compositions and small particle sizes in a single step. In one study, we demonstrated the synthesis of Pt–Ag alloy nanocages through galvanic replacement between Ag nanocubes and a Pt^II^ precursor.^46^ The nanocages exhibited enhanced activity and durability toward oxygen reduction. Interestingly, the composition of the alloy nanocages changed from Ag-rich to Pt-rich upon repeated electrochemical cycling. After 10,000 cycles of accelerated durability test (ADT), the nanocages showed a 3.3-fold enhancement in mass activity relative to that of commercial Pt/C.

**Figure 10:**
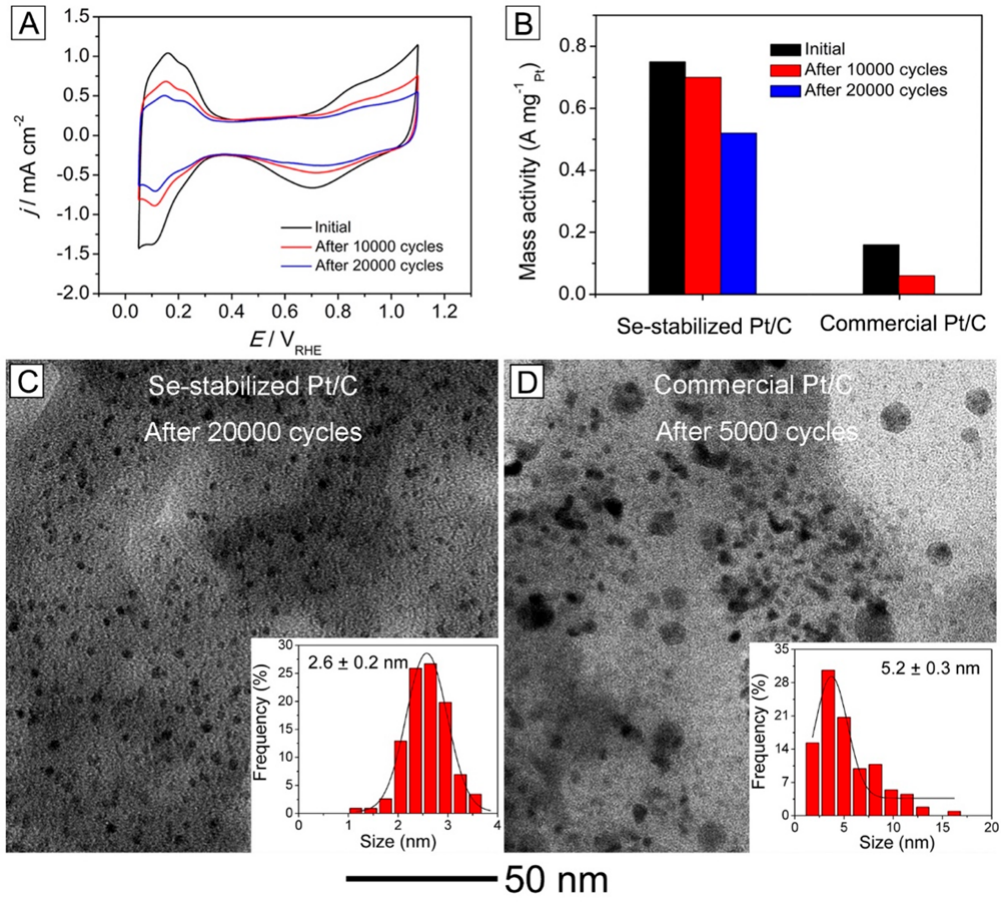
(A) Cyclic voltammetry curves of Se-stabilized Pt/C before and after 10,000 and 20,000 cycles of ADT. (B) Mass activity of the Se-stabilized Pt/C and commercial Pt/C catalysts before and after different cycles of ADT. (C) TEM image of a Se-stabilized Pt/C catalyst after 20,000 cycles of ADT and (D) TEM image of a commercial Pt/C catalyst after 5,000 cycles of ADT. Histograms show the size distributions of the Pt nanoparticles. Reproduced with permission from ref (2). Copyright 2019 American Chemical Society.

In another study, the Se-stabilized Pt/C catalyst derived from the galvanic replacement between Se and Pt^II^ precursor was found to exhibit superior activity and durability toward oxygen reduction.^2^ The residual Se can serve as a linker to strongly anchor the Pt nanoparticles to the carbon support through Pt–Se–C linkage, greatly enhancing the Pt–support interaction and thus improving the catalytic durability. The electrochemical active surface area derived from the charges associated with hydrogen adsorption and desorption was 3.5-times as large as that of commercial Pt/C catalyst (Figure 10A), indicating a substantial reduction in particle size. The stronger Pt–support interaction and stabilization provided by the residual Se atoms further suppressed coalescence while mitigating detachment of the catalytic particles from the carbon support. Even after 20,000 cycles of ADT, the mass activity was still over 3-times greater than the pristine value of commercial Pt/C (Figure 10B). The sizes of the Pt nanoparticles in the Se-stabilized Pt/C after 20,000 cycles of ADT and commercial Pt/C after 5,000 cycles of ADT were 2.6 ± 0.2 and 5.2 ± 0.3 nm, respectively (Figure 10C,D). Most of the Pt nanoparticles in the Se-stabilized Pt/C could be retained on the carbon support without aggregation or detachment. In contrast, significant aggregation and detachment were observed for the Pt nanoparticles in the commercial Pt/C only after 5,000 cycles of ADT.

## 6 Concluding Remarks

We have discussed the rational synthesis of a variety of metal nanostructures with controlled shapes, morphologies, and compositions though galvanic replacement reaction. This approach relies on the oxidation and dissolution of atoms from a substrate and the reduction of a salt precursor, followed by the deposition of newly formed metal atoms on the substrate. The driving force of this reaction is determined by the difference in reduction potential between the two half reactions involved. In the case of a metallic substrate, the initial site for oxidation and dissolution can be controlled by leveraging the facet selectivity and etching capability of a capping agent. The deposited metal can replicate the crystallinity and surface structure of the substrate when the two metals share the same crystal structure and similar lattice constants. In contrast, different nucleation and growth behaviors will be observed when the substrate is made of a nonmetallic material. For example, the deposition of Au atoms on *a*-Se substrate tends to be confined to the original site of nucleation due to the large structure mismatch between crystalline Au and *a*-Se, in addition to the strong binding between Au and Se. Even though core–shell structures could be obtained under certain conditions, the products usually have a polycrystalline structure and a bumpy surface. Although the progression of a galvanic replacement synthesis can be readily followed using a set of techniques, it is still challenging to achieve an atomistic picture of the reaction in real time and under *operando* conditions. The challenge is expected to be overcome though the development of *in situ* characterization tools.
